# Bladder reconstruction using autologous smooth muscle cell sheets grafted on a pre-vascularized capsule

**DOI:** 10.7150/thno.47006

**Published:** 2020-08-20

**Authors:** Hai-Lin Guo, Xu-Feng Peng, Xing-Qi Bao, Lin Wang, Zhi-Ming Jia, Yi-Chen Huang, Jun-Mei Zhou, Hua Xie, Fang Chen

**Affiliations:** 1 Department of Urology, Shanghai Children's Hospital, Shanghai Jiao Tong University, Shanghai 200062, China.; 2 Department of Urology, Shanghai Jiao Tong University Affiliated Sixth People's Hospital, Shanghai 200233, China.; 3 Shanghai Eastern Urological Reconstruction and Repair institute, Shanghai 200233, China.; 4 Department of Urology, Tongji Hospital, Tongji University School of Medicine, Shanghai 200065, China.; 5 Central Laboratory, Shanghai Children's Hospital, Shanghai Jiao Tong University, Shanghai 200062, China.

**Keywords:** tissue engineering, vascularization, cell sheet, prefabrication, bladder

## Abstract

**Rationale:** Construction of functional vascularized three-dimensional tissues has been a longstanding objective in the field of tissue engineering. The efficacy of using a tissue expander capsule as an induced vascular bed to prefabricate functional vascularized smooth muscle tissue flaps for bladder reconstruction in a rabbit model was tested.

**Methods:** Skin tissue expanders were inserted into the groin to induce vascularized capsule pouch formation. Smooth muscle cells and endothelial progenitor cells were harvested and cocultured to form pre-vascularized smooth muscle cell sheet. Then repeated transplantation of triple-layer cell sheet grafts onto the vascularized capsular tissue was performed at 2-day intervals to prefabricate functional vascularized smooth muscle tissue flaps. Bladder muscular wall defects were created and repaired by six-layer cell sheet graft (sheet only), capsule flap (capsule only) and vascularized capsule prelaminated with smooth muscle cell sheet (sheet plus capsule). The animals were followed for 3 months after implantation and their bladders were explanted serially.

**Results:** Bladder capacity and compliance were maintained in sheet plus capsule group throughout the 3 months. Tissue bath stimulation demonstrated that contractile responses to carbachol and KCl among the three groups revealed a significant difference (*p <* 0.05). Histologically, inflammation was evident in the capsule only group at 1 month and fibrosis was observed in sheet only group at 3 months. The vessel density in capsule only and sheet plus capsule group were significantly higher than in the sheet only group at each time point (*p <* 0.05). Comparison of the smooth muscle content among the three groups revealed a significant difference (*p <* 0.05).

**Conclusion:** These results proved that the capsule may serve as an induced vascular bed for vascularized smooth muscle tissue flap prefabrication. The prefabricated functional vascularized smooth muscle tissue flap has the potential for reliable bladder reconstruction and may create new opportunities for vascularization in 3-D tissue engineering.

## Introduction

Tissue engineering is one of the important technologies of regenerative medicine which constitutes and restructures tissue-like construction by the living cells of patient. Since Langer and Vacanti bioengineered cartilage tissue in the shape of an ear by seeding cell suspensions into a three-dimensional (3-D) biodegradable scaffold, scaffold-based tissue engineering has rapidly spread around the world 1-3. This technology has already been used for several clinical applications of bioengineered tissues, including skin, cartilage, bone, and large blood vessels 4-7.

Nonetheless, despite these progresses, new problems still challenge the field. One major barrier impedes effective reconstruction of cell-dense tissues, such as the heart, kidney, and liver 7, 8. In scaffold-based tissue engineering, seeding cell suspensions into porous scaffold materials may fail to produce high cell density and remains problematic. Furthermore, these scaffolds may be gradually replaced with extracellular matrix (ECM) upon biodegradation, producing cell-sparse tissue- engineered constructs with abundant ECM 8, 9. Hence, scaffold-based tissue engineering appears more applicable for ECM-rich tissues, but insufficient for biofabrication of 3-D cell-dense tissues.

Compared with scaffold-based tissue engineering, cell sheet technology features an increased concentration of cells, more uniform cell distribution, and the absence of immunological interference caused by scaffold materials. The preserved 3-D structure of the ECM in cell sheets provides a nutritive and structural microenvironment for cell adhesion and growth, as well as regulating metabolism, differentiation, and regeneration. Moreover, cell sheets can be easily transferred onto damaged organs and 3-D tissues may be engineered by stacking 2-D cell sheets 3, 8, 9. To date, cell sheets have been widely used to fabricate cell-dense functional tissues, and clinical applications include oral mucosal epithelial cell sheets transplanted to repair damage from cornea and esophagus diseases and myoblast sheets implanted to treat severe heart failure 10-12. Both of these technologies have produced native structures alike 3-D tissues.

Another long-standing critical obstacle in tissue engineering is the formation of perfusable blood vessels in 3-D tissue constructs for oxygen and nutrient supplies and waste removal 3, 7, 8. Previous studies proved that thick vascularized tissue can be fabricated by combining cell sheet technology and *in vitro* bioreactor systems 3, 13. Although *in vitro* bioreactors can be designed to mimic *in vivo* microenvironments, duplicating the true *in vivo* conditions under *ex vivo* circumstances is difficult. Furthermore, it does not consider the functional elements of the regenerative environment, including immune, nervous, and hormonal systems, which play crucial roles in tissue regeneration and organ development. An emerging trend to circumvent these problems follows the *in vivo* bioreactor principle, which uses the body as a bioreactor to cultivate the traditional triad (scaffolds, cells, and growth factors) and to leverage the body's own self-regenerative capacity to regenerate new tissue 13-15.

In our previous studies, we have prefabricated a vascularized capsule pouch and proved that it may serve as an *in vivo* bioreactor for thick vascularized smooth muscle tissue flap prefabrication and could also be used as an induced vascular bed for buccal mucosa-lined flap prefabrication for tubularized urethral reconstruction 16-18. We recently developed a simple, cost-effective, and minimally invasive strategy for harvesting pre-vascularized smooth muscle cell (SMC) sheets using a coculture system, in which SMCs were cocultured with endothelial progenitor cells (EPCs) on common polystyrene dishes 19. To investigate the feasibility of using tissue expander capsule as a regenerative niche for thick vascularized smooth muscle tissue flap prefabrication for bladder reconstruction, we transplant pre-vascularized SMC sheets produced from cocultured with EPCs onto the capsule vascular bed for cystoplasty in a rabbit model.

## Materials and Methods

### Animals

**Figure 1:**
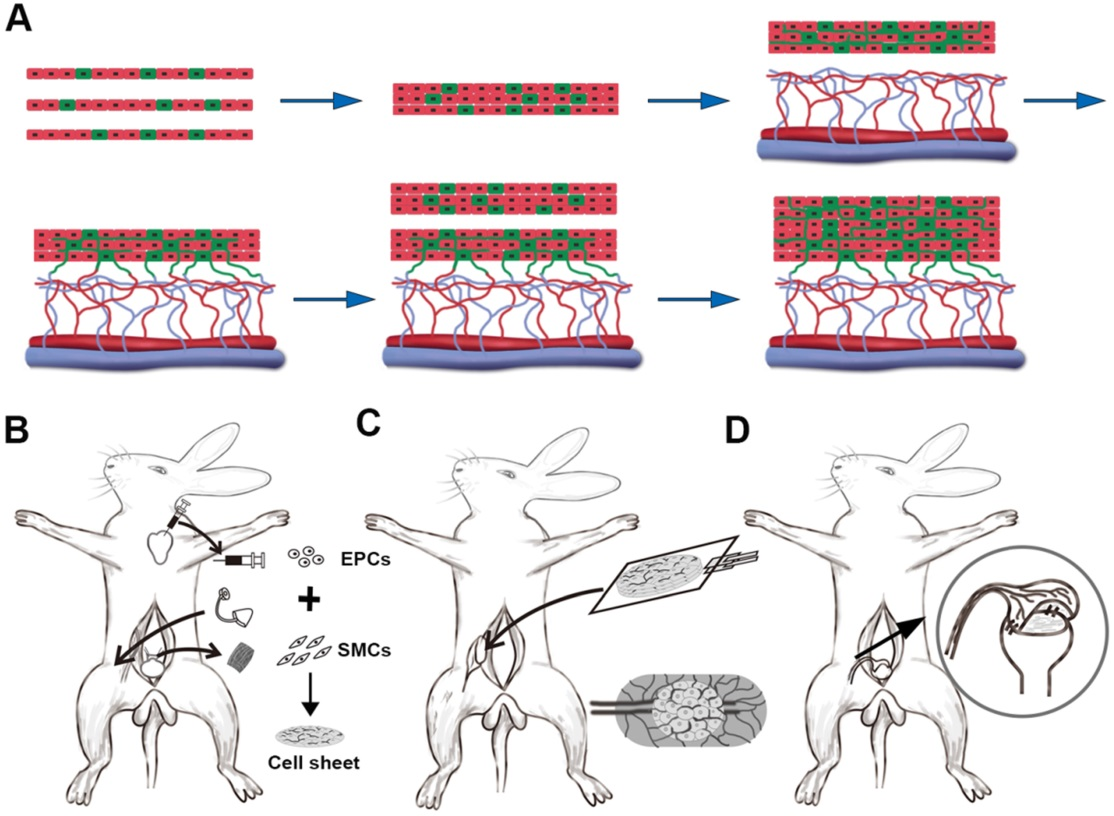
Animal experiment for 3-D vascularized smooth muscle tissue flap prefabrication for bladder reconstruction. (**A**) Three EPCs cocultured smooth muscle cell sheets were stacked as the initial graft and then transplanted onto the capsule vascular bed *in vivo*. After transplantation, the cocultured EPCs formed new blood vessels and connected with the blood vessels that originated from the capsule vascular bed. While sufficient growth of microvascular networks occurred within the first graft, a second triple-layer graft was overlaid on the first. Finally, the multilayer cell-dense tissue construct was perfused through both layers using the underlying vascularized capsule. (**B**) Stage 1, harvesting detrusor and peripheral blood for SMCs and EPCs culture, implantation of the silicon tissue expander into the groin superficial to the SCI vessels to induce vascularized capsule formation and SMCs cocultures with EPCs to form pre-vascularized SMC sheets. (**C**) Stage 2, exploration of induced capsule pouch, explantation of the silicone tissue expander, and transplantion of the layered cell sheets graft onto the capsule vascular bed. (**D**) Stage 3, transposition of the prefabricated capsule smooth muscle composite tissue flap pedicled on the SCI vessels and flap insertion into the bladder muscular wall defects. EPC: endothelial progenitor cell; SCI: superficial circumflex iliac vessels; SMC: smooth muscle cell.

For the 3-stage animal experiments, 40 New Zealand white male rabbits ( stage 1, n = 2; stage 2, n = 14; stage 3, n=24 ) weighing 2.5-3.0 kg (provided by the Department of Laboratory Animal Science, School of Medicine, Shanghai Jiao Tong University) were used (Figure 1). All of the rabbits were anesthetized with an intravenous injection of 20-30 mg/kg sodium pentobarbital and all of the surgical procedures were performed under sterile conditions. The whole animal experimental protocol was approved by the Animal Care and Use Committee of Shanghai Jiao Tong University School of Medicine.

### Stage 1: Cell sheet preparation and capsule vascular bed fabrication

#### Cell culture

SMCs and EPCs were cultured as previously described 18-20. Briefly, a pure detrusor strip approximately 10 × 5 mm was cut down from the bladder wall. Then the free strip was minced and digested to create cell suspensions. The suspensions were seeded at a density of 1 × 10^5^/cm^2^ in smooth muscle growth medium 2 (SmGM-2; Lonza, USA). The SMCs were refed every 2 days until they were 80-90% confluent, usually 6-8 days. SMCs at passages 3 to 5 were harvested for identification or coculture. Myosin heavy chain 11 (Myh 11, mouse monoclonal, Novus Biologicals) and alpha-smooth muscle actin (α-SMA, goat polyclonal, Novus Biologicals) were used for immunofluorescence staining and flow cytometry.

Peripheral blood (20 mL) was obtained via cardiac puncture and EPCs were isolated from other components of the peripheral blood by density-gradient centrifugation with Histopaque-1083, according to the manufacturer's instructions. The cells were then seeded on 24-well plates pre-coated with fibronectin (Sigma, USA) at a density of 1×10^6^ cells/cm^2^ and cultured in microvascular endothelial cell growth medium 2 (EGM-2 MV, Lonza, USA). The medium was half refreshed every day until typical cell colonies formed, usually 10-12 days. EPCs at passages 3 to 5 were used for further experiments.

#### Cocultured cell sheet fabrication and identification

To fabricate pre-vascularized smooth muscle cell sheets, SMCs and EPCs were mixed at a ratio of 6:1 and cocultured on 35 mm polystyrene culture dishes 19. The cells were cocultured in a 1:1 (v/v) mixture of SmGM-2 and EGM-2 MV and the medium was changed every day. To demonstrate proangiogenic biological activity, supernatant of the cocultured cells (SMCs: 6×10^5^/cm^2^, EPCs: 1×10^5^/cm^2^), EPCs (7×10^5^/cm^2^), or SMCs (7×10^5^/cm^2^), after being cultured for 7 days, was collected and centrifuged to remove debris and contaminating cells and levels of vascular endothelial growth factor (VEGF), basic fibroblast growth factor (bFGF), and transforming growth factor-β (TGF-β) in the supernatants were measured using ELISA kits following the manufacturer's instructions. On day 7, CD31 expression within the cocultured cells was detected. Then the cocultured cells were transferred to a biosafety cabinet and a circular scratch was made around the edge of the dish. From the edge to the center, the confluent cell layer was pushed centripetally with a pipette tip and an intact sheet was harvested using mechanical methods (Video S1) 19. The cell viability of the cell sheet before and after harvest was evaluated using a LIVE/DEAD viability/cytotoxicity assay kit (Invitrogen) based on a simultaneous determination of living and dead cells with two probes, calcein-AM for intracellular esterase activity and ethidium homodimer-1 for plasma membrane integrity 21. Immunohistochemical staining, scanning electron microscopy (SEM), and transmission electron microscopy (TEM) were used to observe the morphological features of the harvested cell sheets.

#### Capsule vascular bed fabrication

Tissue expander capsules were induced according to our previously described methods 16-18. Briefly, skin incisions were cut in the bilateral inguinal region and bilateral superficial circumflex iliac (SCI) artery and vein, branches of the femoral artery and vein, were exposed. Then two 15 ml spherical, custom-made silicone tissue expanders were placed superficial to the SCI vessels underneath the bilateral inguinal skin (Figure 1B). After wound closure, the expanders were filled with 3 ml of saline intraoperatively and on days 10, 13, 16, and 19 post-operatively to achieve the final volume of 15 ml. One week after the expanders were fully expanded, the tissue expanders were removed and the vascularized capsules were scrutinized and ready for cell sheet transplantation.

### Stage 2: Vascularized smooth muscle tissue flap prefabrication

#### Stacking cell sheets

To layer the cell sheets, one cell sheet with culture media was gently aspirated into the tip of a pipette and transferred to a new 60 mm polystyrene culture dish. Then a second cell sheet was placed on top of the first and attached by aspiration of the media (Video S2). To ensure the cell sheets obtained adequate nutrition and to avoid the cell sheets detaching from each other, 3 ml medium was added to the 6 cm dish and the culture medium was changed at 10-minute intervals. After incubation at 37 °C for 30 min, triple-layer constructs were finally created using the same procedures (Figure 1A). To visualize the layering structure, the cell sheets were labeled with either a combination of 10 mmol/L CellTracker Red CM-DIL (Invitrogen) or 10 mmol/L CellTracker Green CMFDA (Invitrogen) prior to stacking the cell layers.

#### Transplantation of layered cell sheets over the capsule vascular bed

Cell sheet transplantation was performed according to previously described methods 18, 19. Briefly, triple-layer cell sheet constructs were placed on top of a transparent polypropylene support sheet (3 cm × 2 cm) and transplanted onto the capsule vascular bed by sliding from the nonadhesive polypropylene sheet (Figure 1C). The constructs were stretched on the capsule vascularized by the SCI artery and vein. To maintain as an isolated chamber and prevent adhesion, the water-filled tissue expander was placed back into the capsule pouch and the incisions were closed with 4-0 nylon sutures. Two days later, another triple-layer cell sheets constructs were repeatedly transplanted. For each step, the water-filled tissue expanders were put back into the capsule pouch and the day for the last transplantation was defined as day 0 (D0).

To monitor the survival status of the transplanted cell sheet constructs, 6 rabbits with 12 CMFDA-labeled prefabricated flaps were resected at days 2 after transplantation (3 layers and 6 layers, each n = 6) and 8 rabbits with 16 unlabeled prefabricated constructs were harvested for morphological and physiological analyses 1 month after transplantation (3 layers and 6 layers, each n = 8). The rabbits were then killed with overdoses of pentobarbital.

### Stage 3: Bladder reconstruction with the prefabricated flap

#### Group divisions

Twenty-four rabbits were prepared as described for muscle biopsy 20, 22 and randomly divided into 3 treatment groups with 8 animals each: 1) cell-sheet transplantation (sheet only group), 2) capsule flap (capsule only group), and 3) vascularized capsule prelaminated with smooth muscle cell sheets (sheet plus capsule group). In each group, the bladder neck (far from the biopsy site) was dissected under a surgical microscope and the muscular layer was separated from the urothelium, followed by a partial detrusorectomy with a mean diameter of 20.0 mm.

#### Fabricated 3-D tissue flap transplantation to the bladder

In the sheet only group, a six-layer cell sheet construct with a mean diameter of 20.0 mm was grafted onto the urothelial diverticulum and secured with 6-0 PDS sutures. In the capsule only group, the vascularized capsule (1 month after full inflation) was isolated on its SCI vascular pedicle. After making a small hole in the abdominal wall, the pedicle capsule flap was then pulled from the groin space to the abdominal cavity and wrapped directly onto the bladder diverticulum. In sheet plus capsule group, 6-layer cell sheets were transplanted onto the capsule vascular bed at 2-day intervals as previously described and the prefabricated capsule smooth muscle composite tissue flap (1 month after incubation) was transposed as a pedicled flap into the abdominal cavity toward the bladder muscular wall defect in a similar manner to the capsule only group (Figure 1D). Under 16× microscope magnification, the transposed flap was trimmed, inserted into the diverticulum, and secured with 6-0 PDS sutures. All of the transplants were covered with a fat pad and the fat tissue was sutured to the bladder wall using 6-0 nylon sutures to secure it and act as a marker during the follow-up. Care was taken to avoid penetrating the bladder mucosa during suturing to prevent stone formation. The catheter was left indwelling and fastened with sutures to provide bladder drainage for 14 days post-operation. Prophylactic cefuroxime sodium (0.5 g/day) was administered intravenously for 5 days after surgery.

### Evaluation of the reconstructed bladder

#### Bladder cystometry (urodynamics)

Bladder cystometry was performed according to previously described methods 23. Briefly, the bladder was emptied using manual abdominal pressure. A sterile 8Fr transurethral polyurethane catheter was advanced retrograde into the bladder and connected to a pressure transducer in line with an infusion pump (Duet Logic, Medtronic, Denmark). Saline was infused at 10 mL/min and continuous pressure/flow measurements were recorded (Duet Logic, Medtronic, Denmark). The bladder capacity was defined as the volume of infusion that triggered the first urine leakage. Bladder compliance was calculated using the equation ∆V/∆P, where ∆P (cm H_2_O) was the threshold pressure (pressure that triggered voiding) minus the resting bladder pressure. ∆V was the maximal bladder capacity (mL).

#### *Ex vivo* contractility

Four animals in each group were humanely euthanized 1 and 3 months post-implantation and the regenerated tissue molds harvested from each original implantation site were excised from the native bladders. One half was stored at -80 °C and the second half was placed in oxygenated Krebs solution at 37 °C for organ bath contractility studies. Briefly, regenerated muscle molds were cut into 8 × 3 mm longitudinal tissue strips that were suspended in a modified Krebs solution. The muscle strips were attached to a force transducer, stretched to a preload tension of 1.0 g, and equilibrated for 30 min. Then 80 μM of KCl was used to depolarize the tissue. Dose-response cholinergic contractions were assessed with carbachol at a dosage ranging from 1 × 10^-6^ to 1 × 10^-4^ M. Atropine 10^-4^ M was used as a competitive antagonist 23. Data acquisition software recorded the continuous force displacements.

#### Real-time polymerase chain reaction

Except for histological analysis, the residual samples were flash-frozen in liquid nitrogen and pulverized using a mortar. Total RNA was extracted using an RNeasy Mini kit and the cDNA was synthesized from 1 μg of total RNA using an iScript^TM^ cDNA Synthesis kit (Sangon Biological Engineering, Shanghai, China). The sequence of primers for rabbit Myh 11 was designed as follows: forward, 5'- GCGATTCGGCAAGTTCATCC-3'; reverse, 5'-TGGAAGGTCCTCTCCTCTCG-3'. Reverse transcription was performed at 37 °C for 15 min and qPCR was conducted for 5 min at 95 °C, followed by 40 cycles of 20 s at 95 °C, 20 s at 65 °C, and 20 s at 75 °C. The relative expression level of corresponding mRNA was analyzed using the comparative Ct (^∆∆^Ct) method and normalised by housekeeping gene GAPDH 24.

#### Histological analysis

All of the samples were fixed in 4% paraformaldehyde and embedded in paraffin. Then 5 μm serial sections were generated and evaluated by hematoxylin and eosin (H&E) and immunohistochemistry staining 9,25. Anti-Myh 11 (mouse monoclonal, 1:300 dilution, Novus Biologicals) and anti-α-SMA antibody (mouse monoclonal, 1:500 dilution, Abcam) were used to verify the smooth muscle origin. Anti-CD31 (mouse monoclonal, 1:250 dilution, Novus Biologicals) and anti-CD34 (rat monoclonal, 1:250 dilution, Thermo Fisher Scientific) antibodies were used to verify the vascular origin. The sections were heated in 10 mM Tris-HCl buffer (pH 8.8) for antigen retrieval and the samples treated with primary antibodies were incubated overnight at 4℃. Then the sections were incubated with appropriate secondary antibodies for 1 h at room temperature, followed by subsequent linking to horseradish peroxidase and substrate/chromogen reactions using an immunoperoxidase secondary detection kit (Millipore, Billerica, MA, USA). Negative controls without primary antibodies were prepared to rule out non-specific labeling. The slides were observed using an Axioplan 2 microscope (Zeiss). The positive staining was quantified using Image-Pro Plus 5.1 software (Media Cybernetics, Inc., Rockville, MD, USA) in 8 different fields for each tissue sample.

### Statistical analysis

All of the statistical analyses were conducted using GraphPad Prism 6.0 software (GraphPad Prism, USA). The data were presented as the mean ± SD. Bladder volumes and pressures were recorded as continuous integers. Isometric contractions were recorded as maximal contraction (grams) per individual tissue and normalized to the total tissue weight (g/100 mg). The analysis of variance and Kruskal-Wallis test were used to determine the differences among the three groups at each time points. Differences were considered statistically significant at *p* values <0.05.

## Results

### Characteristics of the SMCs, EPCs, and cocultured cells

**Figure 2:**
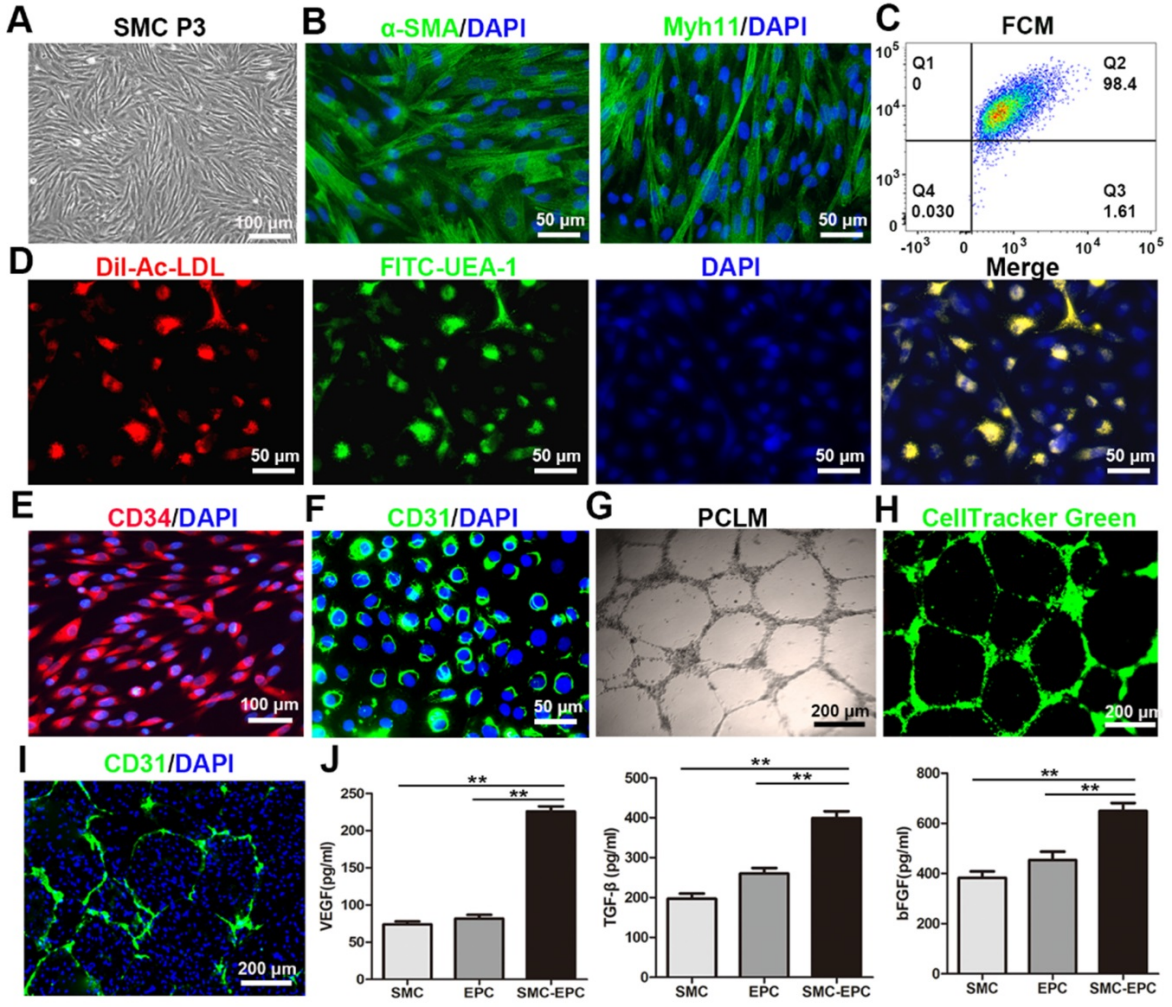
Characteristics of the SMCs, EPCs, and cocultured cells. (**A**) Phase-contrast image of the bladder SMCs at P3. (**B**) The α-SMA and Myh 11 immunofluorescence staining of the SMCs were positive. (**C**) FCM showed that 98.4% of the SMCs were positive for α-SMA and Myh 11. (**D**) The functional characteristics of the EPCs were assessed by measuring their ability to uptake DiI-Ac-LDL (red fluorescence) and bind to FITC-UEA-1 (green). (**E and F**) Immunofluorescence images showing CD34 and CD31 expression in the EPCs. (**G**) Tube formation by the EPCs was assessed on Matrigel-precoated plates using PCLM. (**H**) Tube-forming EPCs were labeled with CellTracker Green (green). (**I**) The networked EPCs within the cocultured cells were stained with anti-mouse CD31 (green). (**J**) Angiogenic factor secretion of the SMCs, EPCs, and SMCs-EPCs cocultured cells was measured via enzyme-linked immunosorbent assay (n = 9). The results are expressed as means ± SD. **p < 0.01. Nuclei were counterstained with DAPI (blue). DiI-Ac-LDL, DiI-acetylated low density lipoprotein; EPCs, endothelial progenitor cells; FCM, flow cytometry; P0, primary culture; P3, third-passage cultures; PCLM, phase-contrast light microscopy; SMCs, smooth muscle cells; UEA-I, Ulex europaeus agglutinin-1.

Bladder SMCs were successfully isolated and exhibited a characteristic spindle morphology at passage 3 (Figure 2A). Immunofluorescence staining and flow cytometry analysis showed that 98.4% of the SMCs were positive for α-SMA and Myh 11 (Figure 2B and C). For primary EPCs, a few cell colonies that demonstrated a typical cobblestone-like morphology appeared after 9-11 days on FN precoated culture plates. After 12-14 days, the cells reached approximately 90% confluence and were sub-cultured. By the third passage (P3), the cultures were relatively homogeneous.

The functional characteristics of the EPCs were identified via ac-LDL endocytosis and UEA-1 binding. The results showed that the EPCs incorporated DiI-Ac-LDL and bound UEA-1 (Figure 2D). Immunofluorescence staining revealed that 98% of the EPCs were positive for stem cell marker CD34 and endothelial marker CD31 (Figure 2E and F). A matrigel network formation assay was performed in both the labeled and unlabeled EPCs. Both the labeled and unlabeled EPCs formed capillary-like tubes with lumens on Matrigel-precoated culture plates (Figure 2G and H).

The SMCs and EPCs in the cocultures grew together in an intertwined manner in the monolayer. At 7 days after coculture, the two cell types were uniformly distributed throughout the monolayer and pre-vascular microcapillary-like networked structures formed in the SMCs-EPCs cocultures on Matrigel-precoated plates (Figure 2I). No such tube-like structures were observed in the SMCs monocultures. Furthermore, the secretion levels of VEGF, bFGF, and TGF-β in the SMCs-EPCs cocultures group were significantly higher (*P <* 0.01) than those in the EPCs and SMCs monoculture group (Figure 2J).

### Characteristics of SMCs-EPCs cocultured cell sheets

**Figure 3:**
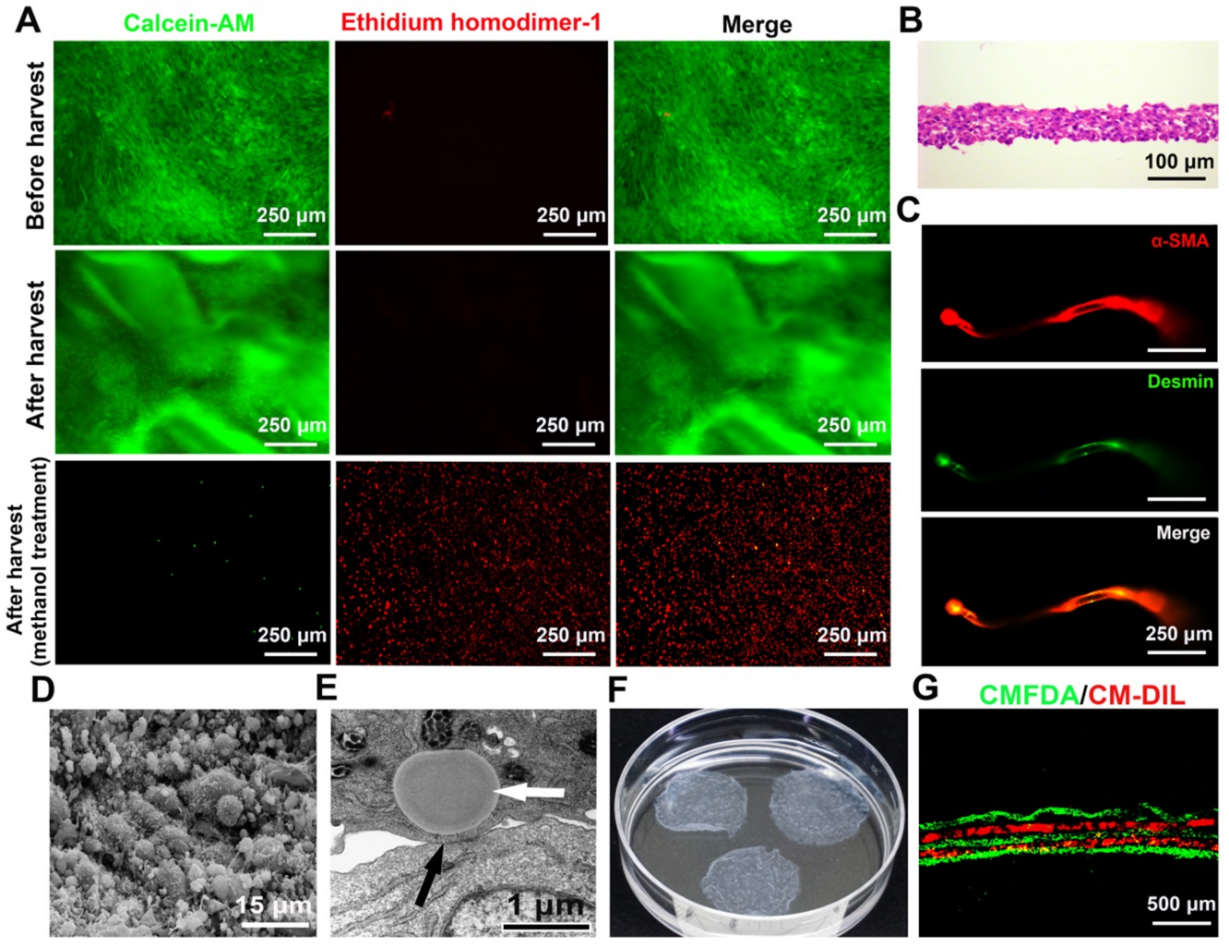
Characteristics of the SMCs-EPCs cocultured cell sheets. (**A**) Live/dead staining of the SMCs-EPCs cocultured cell sheets before and after harvest. This cell sheet-harvesting manipulation gives no damage to the cells. The bottom images of the methanol treatment results are the negative controls. Live cells (green) and dead cells (red). (**B**) H&E staining of the SMCs-EPCs cocultured cell sheets. (**C**) The cocultured cell sheets were stained with α-SMA (red) and anti-desmin (green) antibodies. (**D**) SEM image of the cocultured cell sheets. (**E**) TEM image of the cocultured cell sheet. (**F**) Transferring three-layer cocultured cell sheets to a 6 cm dish. (**G**) Cross-section of the specimen of a stacked-up cocultured cell sheet construct pre-labeled with CMFDA (green) or CM-DIL (red) showing a fine five-layer structure. EPC: endothelial progenitor cell; SEM: scanning electron microscopy; SMC: smooth muscle cell; TEM: transmission electron microscopy.

The cocultured cells became excessively confluent after 7 or 8 days of culture. The cocultured cell sheets were then successfully harvested using a pipette tip within 1 min (Video S1). To determine cell survival upon harvest, the fluorescent stereomicroscopic observation showed that the harvested SMCs-EPCs cocultured cell sheets labeled with calcein-AM and ethidium homodimer-1 had no damaged cells in the cell sheets. As controls, the cocultured cells treated with methanol showed extensively dead cells (Figure 3A). During harvest, the cocultured cells detached together with the ECM and the diameter of the cell sheets shrank to 2.0 ± 0.1 cm (Figure 3F). As a result, the harvested cocultured sheets consisted of 4 to 7 cell layers, and the thickness of the cell sheets was 60.0 ± 6.3 μm (Figure 3B). Immunofluorescent staining showed that the cocultured cell sheets expressed typical SMCs markers, such as a-SMA and desmin (Figure 3C).

The ultrastructure of the cocultured cell sheets was examined by SEM and TEM. In the SEM analysis, the surface of the cocultured cell sheets was covered with abundant ECM with microsphere-like morphology, while microvilli were distributed on the cell membrane (Figure 3D). In the TEM analysis, gap junctions were observed between cells and lipid droplets were observed in the cytoplasm of the SMCs (Figure 3E), which were similar to the structures observed in the native bladder. Moreover, the cocultured cell sheets could be stacked up one by one to form a 3-D tissue construct (Video S2). To confirm the layering structure, the cocultured cell sheets were pre-labeled with CellTracker CM-DIL (red) or CellTracker CMFDA (green), and a stacked-up construct was prepared by layering the former and latter labeled sheets alternately. The fluorescence images of the cross-section of the construct showed a fine five-layer structure that had two red and three green layers alternating (Figure 3G).

### Macroscopic and histology analysis of the vascularized capsule

**Figure 4:**
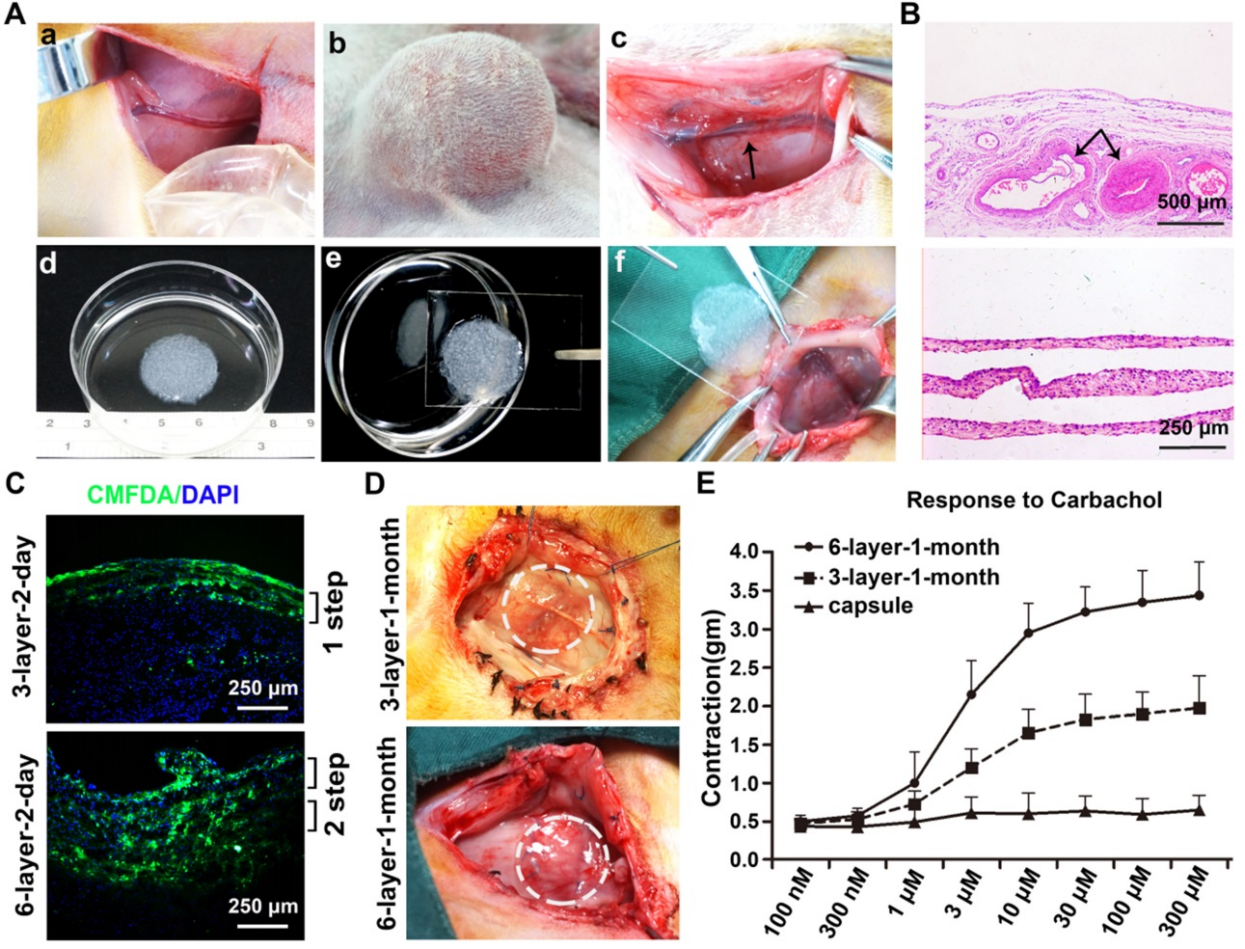
Fabrication of viable vascularized smooth muscle tissue by transplanting stacked cell sheets on the induced capsule vascular bed. (**A**) Vascularized capsule induction and stacked cell sheet transplantation. a) the tissue expander was placed close to the separated SCI vessels; b) groin exploration 1 week after the expander was fully inflated with saline solution; c) the tissue expander was removed and the induced vascularized capsule with axial vessels was exposed; d) three-layer cell sheets were stacked as the initial graft; e) the stacked cell sheets were transferred on a transparent polypropylene support sheet; f) the sheets were transplanted on the capsule vascular bed. (**B**) H&E staining of the induced capsule and stacked cell sheet graft. (**C**) Immunofluorescence staining of the CMFDA (green) labeled cell sheets transplanted on the capsule vascular bed for 2 days. Nuclei were counterstained with DAPI (blue). (**D**) Macroscopic view of the transplanted cell sheets on the capsule vascular bed for 1 month. The dashed lines indicate the transplanted cell sheet graft. (**E**) Dose-response curves of carbachol in the transplanted cell sheets on the capsule vascular bed for 1 month. Arrows, the superficial circumflex iliac vessels; 3-layer-2-day, 3-layer cell sheets transplanted on the capsule for 2 days; 6-layer-2-day, 6-layer cell sheets transplanted on the capsule for 2 days; 3-layer-1-month, 3-layer cell sheets transplanted on the capsule for 1 month; 6-layer-1-month, 6-layer cell sheets transplanted on the capsule for 1 month.

A macroscopic vascularized capsular tissue in the shape of a hollow viscus with a smooth surface was induced by tissue expansion (Figure 4A). The SCIs were located in the center of the capsules. As axial vessels, the SCIs remained pulsatile, and numerous small vessels were observed originating from the axial vessels and extending to the periphery of the capsules. Pathologic studies further confirmed the evidence of neovascularization within the capsules, and abundantly impregnated vascular structures near the SCI vessels were observed with parallel developed collagen fibers (Figure 4B).

### Outcome of stacked cell sheets transplanted onto the vascularized capsules

CMFDA, a green-fluorescent dye well suited for monitoring cells, was used to track the survival status of the transplanted cell sheets. For the 3-layer and 6-layer CMFDA-labeled cell sheets transplanted onto the vascularized capsules, fluorescence microscopy of the cell sheets showed that they were successfully transplanted onto the vascularized capsules and almost all of the grafted cells were alive 2 days after transplantation without infection (Figure 4C).

**Figure 5:**
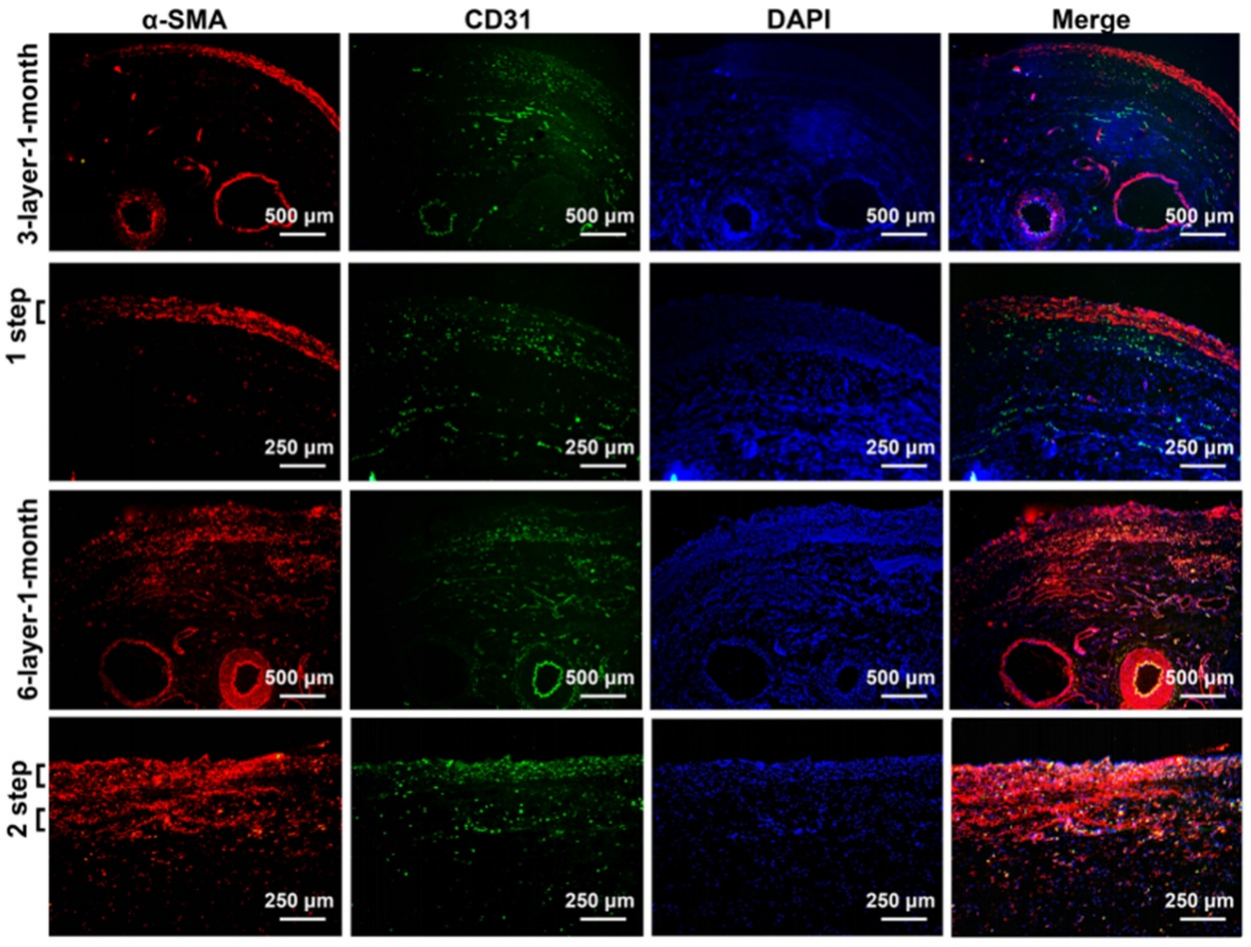
Immunofluorescence analysis of the cell sheets transplanted on the vascularized capsule for 1 month. Fluorescence microscopy revealing smooth muscle tissue labeled with red fluorescence (α-SMA) and micro-vessels and axial vessels within the composite tissue labeled with green fluorescence (CD31). DAPI staining identifies all of the cell nuclei (blue). 3-layer-1-month, 3-layer cell sheets transplanted on the capsule for 1 month; 6-layer-1-month, 6-layer cell sheets transplanted on the capsule for 1 month.

After 1 month of incubation, the transplanted cell sheets formed macroscopically viable tissues that could be easily distinguished from the surrounding capsular tissue (Figure 4D). Capillary vessel extensions into the overlying cell sheet grafts were evident and the SCIs remained pulsatile. The composite tissue flap was raised from the abdominal wall and the final viable tissue was harvested for functional and histological analysis. The functional regenerations of the composite tissue were assessed through contractile responses to carbachol, and the 6-layer constructs displayed higher contractile responses to carbachol than the 3-layer constructs (Figure 4E). Immunofluorescence staining further confirmed that the double-step transplantation of two triple-layer grafts at 2-day intervals permitted whole tissue survival with a well-organized microvascular network, and the two grafts became intimately connected, resulting in a thicker smooth muscle tissue than one triple-layer graft (Figure 5). Multiple transplantation of the triple-layer cell sheets on the vascularized capsules produced thick functional tissues with perfusable blood vessels under *in vivo* condition.

### Physiologic bladder testing

**Figure 6:**
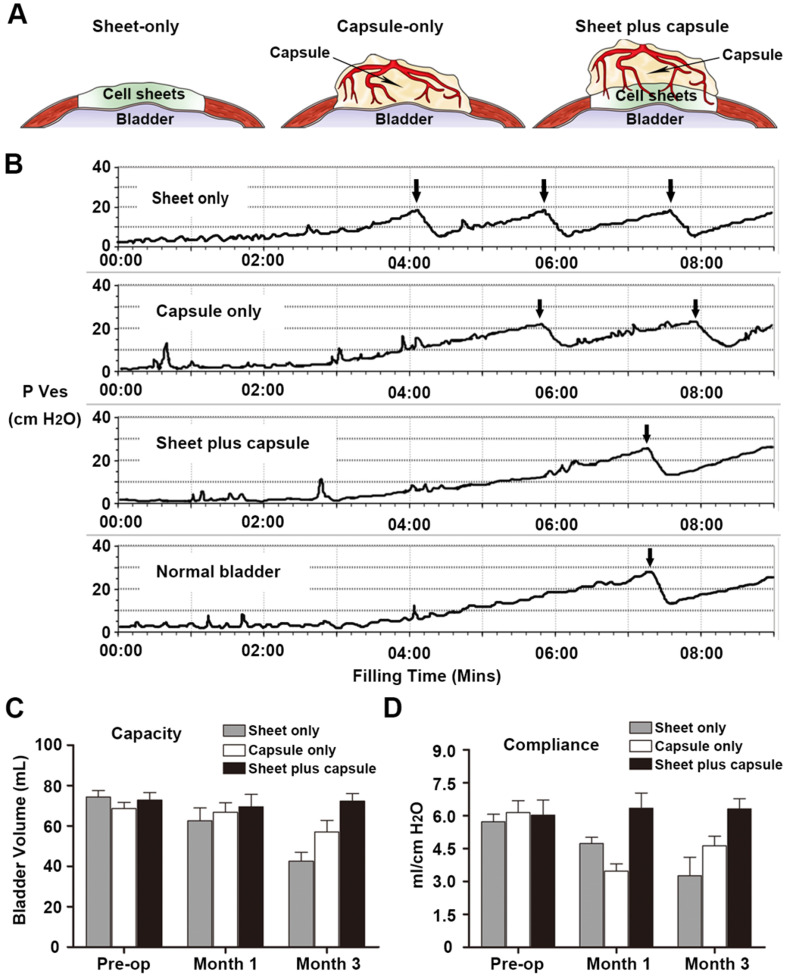
Cystoplasty and physiologic bladder testing. (**A**) Procedural schemes for treatment groups. (**B**) Pressure/volume curves in each group 3 months after cystoplasty. (**C**) Comparison of bladder capacity and (**D**) compliance at each time point in the three groups. Bladders repaired with the prefabricated capsule smooth muscle composite tissue maintained superior volume and compliance over 3 months.

All of the rabbits survived until their scheduled time points and cystometric bladder measurements, which consisted of continuous filling pressure and volume measurements in the rabbit bladders, were recorded in all of the animals without complications. The mean rabbit bladder capacity prior to surgery was 71.83 ± 4.00 mL and the mean bladder compliance was 5.96 ± 0.53 mL/cm H_2_O. At 1 month post-implantation, the bladder capacities were not different among the groups. In contrast, the bladder compliance worsened at this time point in the sheet only and capsule only groups. By 3 months, the bladder capacity and compliance decreased in the sheet only and capsule only groups. Only the bladders repaired with vascularized capsule-smooth muscle composite tissue flaps (sheet plus capsule) maintained normal bladder capacities and compliance throughout the study (Figure 6).

### Gross view and* ex vivo* contractility of the retrieved bladders

**Figure 7:**
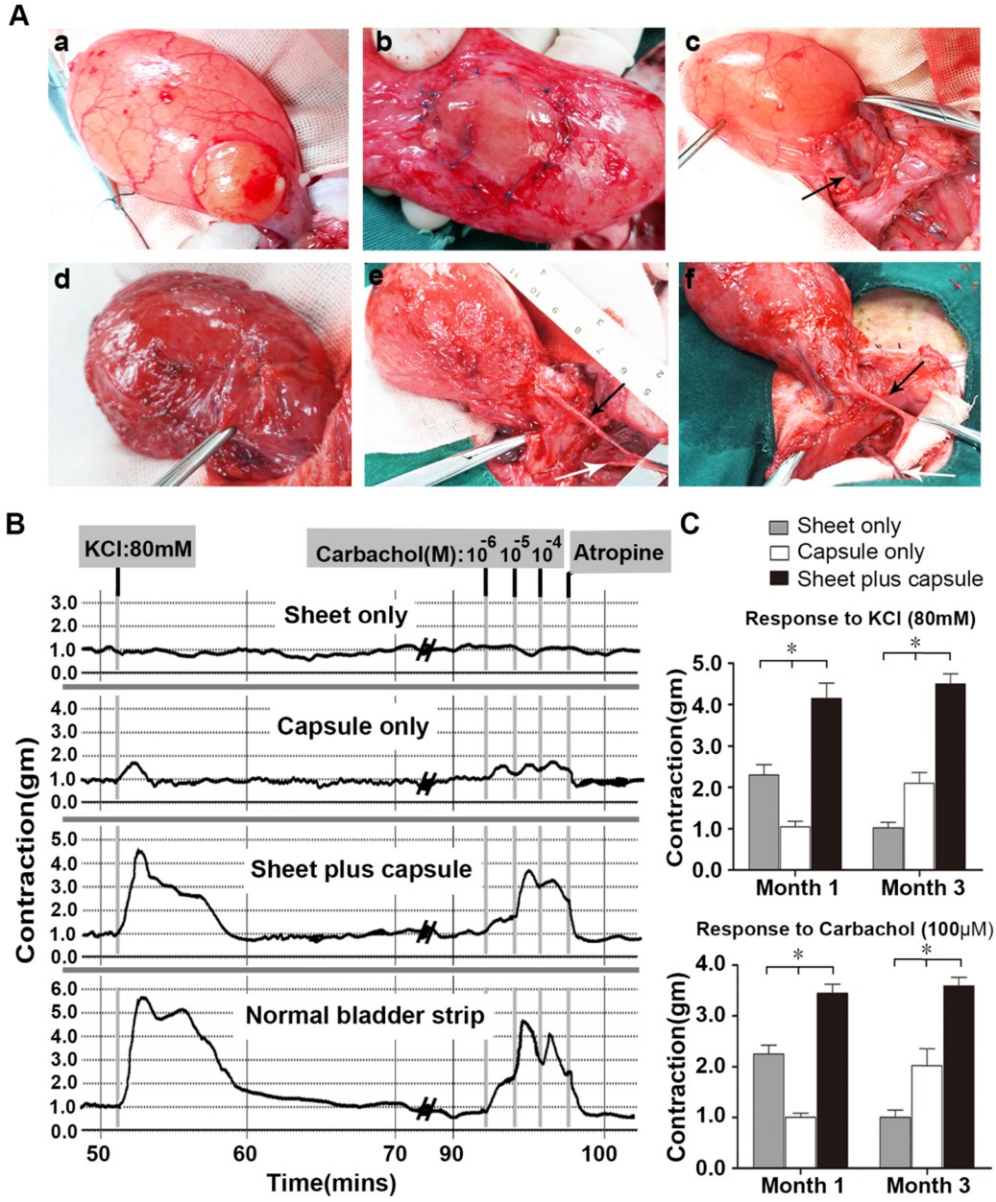
Augmentation cystoplasty and pharmacologic smooth muscle contraction. (**A**) Surgical technique and macroscopic examination of reconstructed bladder wall 3 months post-operatively. a) Partial detrusorectomy. b) Grafting of the layered cell sheet construct on the urothelial layer. c) Transposition of the prefabricated capsule smooth muscle composite tissue flap onto the urothelial diverticulum. Urinary bladders augmented with the layered cell sheets (sheet only; d), the capsule flap (capsule only; e), and the prefabricated capsule smooth muscle composite tissue flap (sheet plus capsule; f) 3 months after bladder muscular wall defect reconstruction. In the sheet only group, fibrosis and contracture of bladders were observed. In the capsule only and sheet plus capsule groups, the axial vessel remained pulsatile and the bladder muscular wall defect was nicely reconstructed by the flap. (**B**) Carbachol (1 x 10^-6^ M to 1 x 10^-4^ M) and KCl (80 mM) tissue bath recordings from a normal rabbit bladder, cell sheet graft, capsule flap, and the prefabricated capsule smooth muscle composite tissue flap 3 months after cystoplasty. (**C**) Contractile responses to KCl (80 mM) and carbachol (100 µM) at each time point in the three groups. * Significant difference among the three groups at each time point after cystoplasty (*p <* 0.05). The black arrow, the superficial circumflex iliac vessels; the white arrow, the femoral vessels.

All of the rabbits were able to void spontaneously after surgery and there were no signs of infection or bladder calculus at all of the follow-up times. The capsule flaps and capsule-smooth muscle composite tissue flaps were long enough for bladder muscular wall reconstruction and none of the flaps needed reconfiguration. In the sheet only group, fibrosis and shrinkage were observed at each time point. In contrast, gross examination at retrieval in the capsule only and sheet plus capsule groups demonstrated normal-appearing tissue without any evidence of fibrosis or scarring at the end of 3 months (Figure 7A).

Tissue strips with the same size, as determined by the wet tissue weight, were used for smooth muscle contractility in an oxygenated Krebs solution organ bath. In the sheet only group, the retrieved bladder strips showed mild KCl and carbachol induced contractions at 1 month, but contractions were barely observed at 3 months. In contrast, the capsule-repaired bladders (capsule only) showed no evident responses to KCl and carbachol stimulation at 1 month, but demonstrated mild responses to KCl and carbachol at 3 months. At 1 and 3 months, the cell-seeded composites group (sheet plus capsule) displayed similar contractile responses to carbachol and KCl, which were reversible with atropine (Figure 7B). Comparisons of the contractile responses to carbachol and KCl among the three groups 1 and 3 months after surgery revealed a significant difference (*p <*0.05; Figure 7C).

### Molecular and histopathologic analyses of retrieved bladders

**Figure 8:**
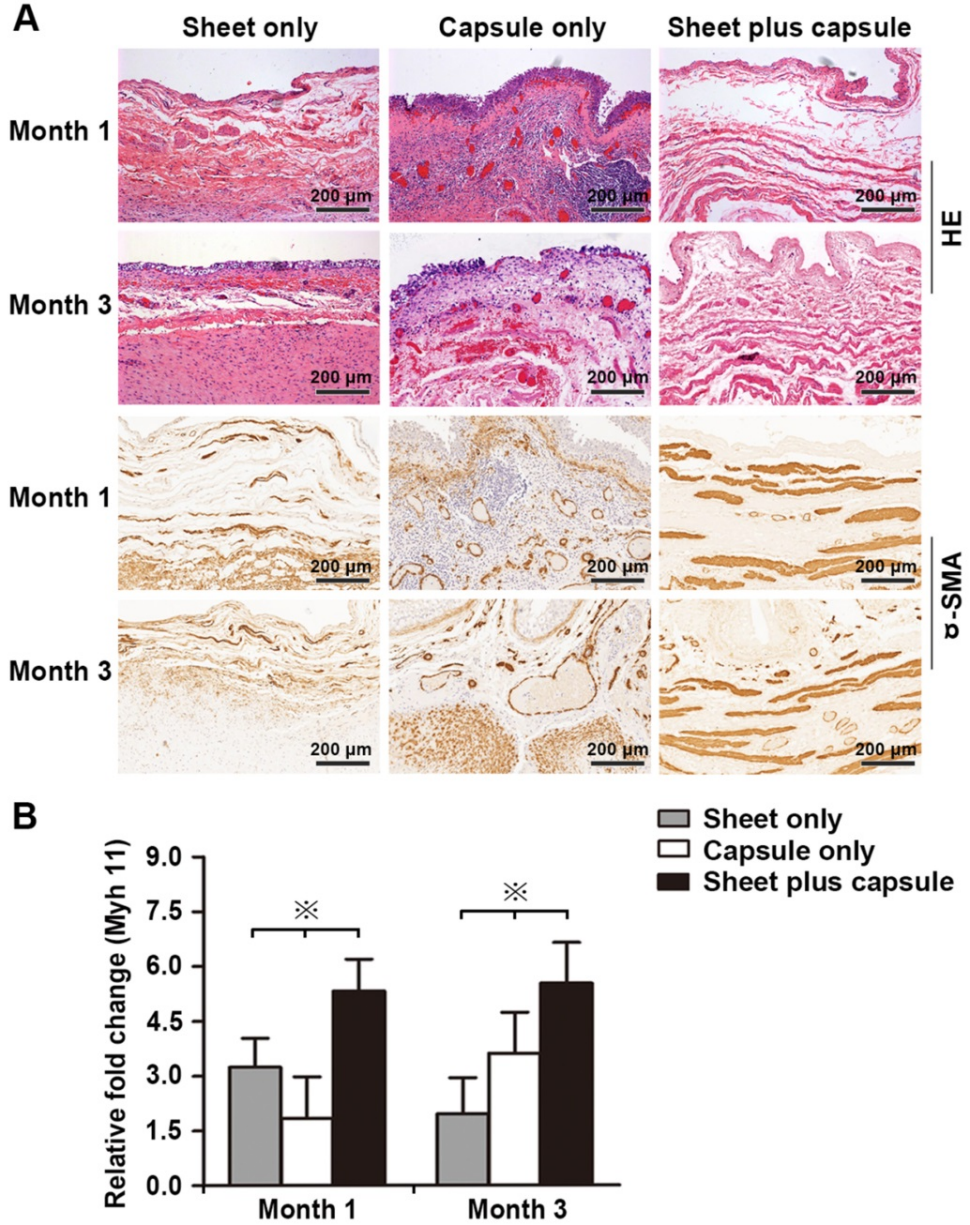
Molecular and histological analysis of reconstructed rabbit bladders. (**A**) Representative H&E and α-smooth muscle actin (α-SMA) immunohistochemical staining in retrieved bladders 1 and 3 months after cystoplasty in the 3 groups. (**B**) Induction of the smooth muscle myosin heavy chain 11 (Myh 11) expression in newly formed tissues was determined by real-time polymerase chain reaction. Values were normalized to the GAPDH levels. ※ Significant difference among the three groups at each time point after cystoplasty (*p <* 0.05).

**Figure 9:**
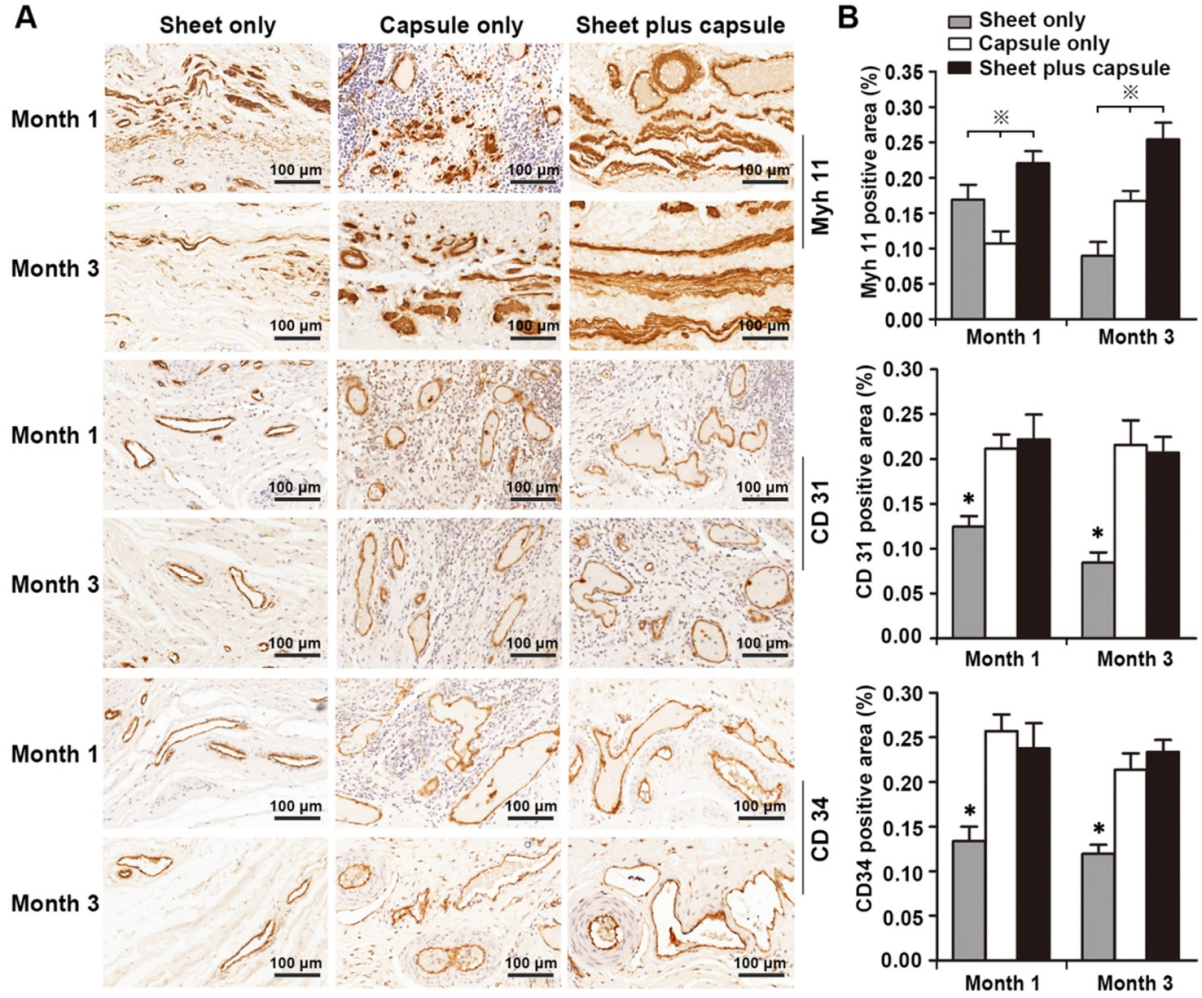
(**A**) Immunohistochemical staining to assess smooth muscles and vessels in the retrieved bladders 1 and 3 months after implantation in the three groups. (**B**) Image analysis of the smooth muscle content and endothelium density at each time point in the three groups. ※ Significant difference among the three groups at each time point after cystoplasty (*p <* 0.05). * Significantly lower than the capsule only and sheet plus capsule groups.

RT-PCR was used to test the gene expression level of the smooth muscles and H&E and IHC staining were used to assess the amount of smooth muscle and neovascularization in the epithelial lower layer. In the sheet only group, disorganized smooth muscle tissue was observed 1 month after surgery. At 3 months, the bladder muscular wall defects were filled with dense fibrous tissues and there was little evidence of smooth muscle and neovascularization surrounding the urothelium. In the capsule only group, scattered discrete smooth muscle tissue and progressive infiltration of inflammatory cells were identified on the retrieval bladder constructs 1 month after surgery. At 3 months, the smooth muscle became more organized and intensive and vascular proliferation was evident beneath the urothelium. In the sheet plus capsule group, bundles of intensive organized smooth muscle tissues with minimal inflammatory cells were observed on the retrieved bladder muscular wall constructs 1 and 3 months after surgery, which could be clearly distinguished from the sheet only and capsule only groups (Figure 8A and Figure 9A). Comparison of the gene expression level of Myh 11 and the amount of smooth muscle tissue among the three groups 1 and 3 months after surgery revealed a significant difference (*p <* 0.05; Figure 8B and Figure 9B). The endothelium density in the capsule only and sheet plus capsule groups was significantly higher than in the sheet only group 1 and 3 months after surgery (*p <*0.05; Figure 9B).

## Discussion

The field of tissue engineering has progressed rapidly, but poor vascularization remains a major obstacle in bioengineering cell-dense tissues, limiting the viable size of constructs due to hypoxia, nutrient insufficiency, and waste accumulation. Prior classical vascularization strategies focused on stimulating vascular ingrowth into tissue constructs, which can be achieved by incorporating growth factors or endothelial cells 26-28. However, these so-called angiogenic approaches are inadequate because the average growth rate of newly developing micro-vessels is only ~5 μm/h 7, 29. Thus, the complete vascularization of large implants after transplantation requires a prolonged time period that is associated with major tissue loss due to hypoxic conditions. In the present study, we fabricated functional 3-D smooth muscle tissues with perfusable blood vessels by combining cell sheet-based tissue engineering and capsule induction techniques. These strategies allow free layered cell sheet grafts to be transferred as pedicled flaps for bladder muscular wall reconstruction and have a major advantage that the prefabricated flap is directly reperfused after implantation at its final destination. These cocultured cell sheet integration methods and flap prefabrication technique may overcome long-standing barriers to producing thick, vascularized tissues and may have potential applications for the treatment of tissue defects and organ failure.

Cell sheet engineering, which has a major advantage of allowing the retrieval of intact cell layers along with their naturally organized extracellular matrix (ECM), has emerged as a promising strategy for generating pre-vascularized cell-dense tissues without requiring any scaffold materials 7, 21, 30. In this study, we fabricated pre-vascularized SMC sheets based on a cocultured system in which SMCs were cocultured with EPCs at a ratio of 6:1. The cocultured EPCs developed a sprouting appearance rather than the classic cobblestone morphology within the smooth muscle sheet. Furthermore, due to the increased secretion of angiogenic growth factors, the cocultured cell sheets containing EPCs appeared to possess a significant innate potential for neovascularization even before transplantation. After the formation of capillary-like networks, the smooth muscle cell sheets could be harvested without disruption from common polystyrene dishes. Using mechanical cell sheet harvest without enzymatic digestion, the previously established endothelial networks within the cocultured cell sheets could be maintained, allowing for the rapid development of graft-derived vascular structures into mature micro-vessels upon transplantation to the host vascular bed.

The capsule induction technique provides several essential advantages for vascularization in tissue engineering. The silicone block or expander (for application in larger animals or humans) creates a spherical capsule pouch and the induced foreign body reaction provokes a predictable neovascularisation in the capsule tissue 31-33. By placing the expander in the vicinity of large vessels, neovascularization with improved blood flow and extensive vascular plexus in the capsule can be controlled and directed, so that the later-induced capsule pouch will be supplied by a single vascular pedicle. This procedure is also known as flap prefabrication, which is occasionally used in plastic and reconstructive surgery. The fact that the capsule flap was consistently viable after transposition indicates that this tissue-induction technique is a safe and reliable procedure. Any pedicle suitable for a later transfer and that can be sacrificed without distal tissue compromise can be used for this prefabrication procedure. Although the capsule predominantly contains fibrous tissue, it has been shown that during the initial stage of the foreign body reaction, a highly increased vasculature around the vascular pedicle can be proven, which supports grafting provided a suitable cell delivery vehicle is used.

Previous studies proved that fibrin glue is an essential supplement for seeding autologous cells onto induced vascularized capsules 31-33. However, transplanted single cells lack cell-to-cell communications and cannot produce an evenly distributed urothelium lining on vascularized capsules. In this study, we cocultured SMCs and EPCs to form intact, viable, and pre-vascularized SMC sheets. The sheets contained ECM, preserved cell-to-cell connections, and were stacked on each other or transferred onto the vascularized capsule without any auxiliary supplements. Furthermore, the transplanted pre-vascularized SMC sheets survived and were evenly distribute on the vascularized capsules. Due to the multiple transplantation of triple-layer cell sheets, the vascular network within the pre-vascularized SMC sheets connected to the capsule vasculature through anastomoses, allowing for direct blood flow between the engineered tissues and host capsule. The transplanted sheets ultimately formed vascularized functional smooth muscle tissues, and the thickness and contractions in response to the muscarinic agonist carbachol increased linearly in accordance with the number of layered cell sheets, demonstrating that 2-day intervals are viable for neovascularization within the first triple-layer cell sheets and perfusion from the axial vessels is sufficient for whole-transplant survival.

Autologous SMC sheet grafting has the potential for reliable bladder reconstruction. The retention, survival, and engraftment of transplanted cells in cell sheet therapy are largely influenced by the degree of vascularization in the transplanted area, and lack of sufficient vasculature limits the viable tissue thickness to 3 layers 8, 18. In the sheet-only group, 6 layered pre-vascularized SMC sheets were directly transplanted onto the bladder muscular wall defects one at a time. However, the lack of sufficient vascularization in the urothelial diverticulum limited the viability and thickness of the layered SMC sheets, resulting in decreased capacity and compliance over time. In the capsule-only group, the capsule flap contained abundant vascular networks and it was easily patched onto the bladder urothelial diverticulum. Although inflammation was evident within the grafted patch 1 month after grafting, the ingrowth of native bladder smooth muscle into the capsules over time was observed by histological examination. The muscle bundles appeared to arise from the normal bladder edges, and the results were consistent with previously reported studies 23, 34. However, the small disorganized smooth muscle bundles in the sheet-only and capsule-only groups were not sufficient to maintain normal bladder capacity and compliance. In the sheet plus capsule group, the vascularized capsule provided adequate nutrition for the SMC sheets to survive and grow on the bladder urothelial diverticulum. Bladder augmentation with the capsule and smooth muscle composite tissue maintained their initial bladder capacities and compliance for the 3 months of the study. These results proved that vascularization is vital for the survival and remodeling of transplanted cells and may have some rules in reconstructed tissues.

Bladder compliance represents the bladder's ability to expand while maintaining a safe low bladder pressure and a well-organized muscular layer can improve bladder compliance. Poorly compliant bladders do not stretch and require high pressures to expand. As they fill under high bladder pressures, these bladders can cause kidney damage. According to Figures 6, 7, 8, and 9, bladder compliance was consistent with the smooth muscle tissue volume. We found that bladder compliance decreased at 1 month in the sheet only and capsule only groups, but bladder capacity showed no significant changes at this time. Thus, bladder compliance was more sensitive to local defects in the bladder smooth muscle tissue than bladder capacity. In the short term, repaired bladders may have little impact on bladder capacity, but bladder capacity may decrease over time. Functional vascularized smooth muscle tissues are vital for maintaining normal bladder capacity and compliance.

In the clinical replacement of many internal organs, direct multistep transplantation of bioengineered tissues may be challenging because each individual procedure has a relatively high risk of complications. Alternatively, ectopic fabrication of viable thick tissues and their transplantation into target organs may alleviate complications 7, 13, 14. In this study, we fabricated viable thick tissues perfused via surgically accessible and connectable blood vessels using an existing host artery and vein. The arteriovenous bundle permits the successful transplantation of ectopically bioengineered cell-dense tissues and helps avoid post-transplant vascularization. These combined techniques for *in vivo* tissue engineering may be applied to many other tissue types in addition to the smooth muscle tissue demonstrated herein and may facilitate the discovery of new biological and medical techniques as well as lead to further advances in tissue engineering and its potential therapeutic applications.

Although this study's results are encouraging, it had some limitations. The major disadvantage lies in the multiple surgical interventions and long development cycles required to fabricate vascularized smooth muscle tissue flaps. Furthermore, bladder wall reconstruction always requires vascularized composite tissue that contains smooth muscle bundles and epithelial lining, so the production of a consistent bilayer structure appears more applicable for bladder augmentation. In our subsequent series studies, we are attempting to fabricate bionic vascularized composite tissue that contains smooth muscle bundles and epithelial lining for bladder augmentation. Moreover, the bladder muscular wall defects and number of experimental animals that were evaluated per time point is low and the results only represent short-term success. A larger sample with more bladder wall defects and a longer-term follow-up would be more convincing. In addition, we merely explored the possibility of three-layered SMC sheets, while the maximum layer of SMC sheets that could be successfully transplanted on the axial capsule vascular bed at a single time was not investigated. When the thickness limitation is overcome, cell sheet engineering will provide groundbreaking treatment options for injured tissues or organs.

## Conclusions

The present step-by-step polysurgery approach based on cell sheet integration and capsule induction technique appears feasible for fabricating viable thick vascularized tissues with perfusable blood vessels. This prefabricated flap may be safely transposed with its new pedicle for bladder muscular wall reconstruction. This technique may create new opportunities for *in vivo* tissue engineering and the clinical repair of various damaged organs.

## Supplementary Material

Supplementary video/movie S1.

Supplementary video/movie S2.

## Abbreviations

3-D: three-dimensional
ECM: extracellular matrix
EPC: endothelial progenitor cell
H&E: hematoxylin and eosin
IHC: immunohistochemistry staining
SCI: superficial circumflex iliac vessels
SEM: scanning electron microscopy
SMC: smooth muscle cell
TEM: transmission electron microscopy
